# Overcoming Subacromial Impingement, as a Term and Concept: Rationale and Proposals

**DOI:** 10.3390/jcm15197356

**Published:** 2026-09-22

**Authors:** Federico Sonnati, Enrico Visonà, Giovanni Merolla

**Affiliations:** 1 AZIMUT Riabilitazione, 13900 Biella, Italy; federico.sonnati@gmail.com; 2 Casa di Cura Villa Maria, 35138 Padova, Italy; enricovisona@icloud.com; 3 Shoulder and Elbow Unit, AUSL Romagna, 47841 Cattolica, Italy; 4 Center of Arthroscopy, Upper Limb Trauma and Minimally Invasive Surgery, Nova Clinic, 47895 Domagnano, San Marino; 5 Biomechanics Laboratory, AUSL Romagna, 47841 Cattolica, Italy

**Keywords:** shoulder pain, shoulder impingement, rotator cuff

## Abstract

**Background/Objectives**: Subacromial impingement syndrome has traditionally been described as pathological contact between the rotator cuff and the anterolateral aspect of the acromion and has long been regarded as a primary cause of shoulder pain and tendon injury. Despite a substantial body of literature questioning the very existence of subacromial impingement as a pathological entity, the diagnosis continues to be widely used in medical and rehabilitation settings as a predominantly biomechanical explanation for shoulder pain. The objective of this review is to determine whether the concept of subacromial impingement should be abandoned in clinical practice, interprofessional communication, and the clinician–patient relationship, and to explore which alternatives may be more appropriate. **Methods**: This narrative review is based on an appraisal of the most relevant literature on this topic published in recent years using scientific databases. **Results**: Evidence suggests that narrowing of the subacromial space is a consequence of rotator cuff muscle–tendon unit incompetence rather than its cause. Furthermore, such dysfunction appears to arise primarily from degenerative rather than compressive mechanisms. Advances in shoulder biokinematics have also challenged the validity of the clinical ‘special tests’ traditionally used to support the diagnosis, as these tests do not appear to accurately identify the presumed target structures. Moreover, surgical decompression of the subacromial space has not proved to be more effective than conservative treatment when performed as a standalone procedure, and it often provides no additional benefit when combined with rotator cuff repair in terms of reducing re-tear rates. Nevertheless, it may still have a role as an adjunctive procedure to improve exposure of the surgical site or in selected cases. **Discussion**: An inaccurate biomechanical explanation may lead patients to believe that arm elevation is inherently harmful, thereby resulting in disability and increased pain. It may also encourage the selection of invasive treatments over conservative management, despite current international guidelines recommending conservative treatment as the first-line approach for subacromial shoulder pain. Indeed, such an approach has been shown to be effective in a substantial proportion of patients, with lower costs and risks, less time away from work, and the advantage that it does not preclude surgery if subsequently required. **Conclusions**: The concept of subacromial impingement appears to be flawed, as the biomechanical mechanism on which it is based is not supported by current evidence. This concept should therefore be reconsidered in clinical practice because of its potential nocebo effects on patients and its capacity to divert clinicians from more appropriate clinical assessment and management strategies. Accordingly, this review proposes alternative terminology and conceptual frameworks better aligned with current evidence and contemporary approaches to the assessment and management of subacromial shoulder pain.

## 1. Introduction

Subacromial impingement syndrome (SIS) refers to pathological contact between the rotator cuff and the anterolateral aspect of the acromion. During arm elevation or abduction, this pathological contact—thought to result from altered acromial morphology, abnormal scapular motion, or impaired activation of the muscles acting on the glenohumeral joint—has traditionally been considered a primary cause of shoulder pain and tendon lesions. According to the available literature, subacromial impingement syndrome (SIS) accounts for 44–65% of shoulder pain diagnoses, with a 30–40% rate of unfavourable outcomes two years after treatment [1,2]. The therapeutic approaches traditionally proposed—including strengthening the shoulder extensor muscles to reduce superior migration of the humeral head [3], modifying scapular motion [4], or alternatively performing acromioplasty to increase the subacromial space—are based on structural and biomechanical concepts supported by low-quality evidence and underpinned by a questionable theoretical construct. Specifically, Neer’s extrinsic theory, first proposed in 1972 [5] and based on anatomical and radiographic observations, postulates that the bursal surface of the supraspinatus tendon progressively abrades against the coracoacromial arch, with the extent of damage depending on acromial morphology (flat, curved, or hooked). This theory has been widely challenged, particularly by contemporary literature.

Despite numerous authoritative publications strongly questioning the very existence of subacromial impingement syndrome [1,6,7,8], the diagnosis continues to be used in both medical and rehabilitation settings as a predominantly biomechanical, structure-focused explanation for shoulder pain. Furthermore, the body of scientific literature addressing the treatment of this condition continues to grow, exploring methods to increase the subacromial space or reposition the humeral head [9,10] and using clinical tests with low discriminatory power as a rule of thumb to support the presence of such compression [11], thereby explaining subacromial pain as a purely biomechanical condition characterised by a reduction in the subacromial space.

At the same time, publications questioning SIS do not incorporate the most recent evidence.

The aim of this narrative review is to summarise the latest knowledge on the subject from new and broader perspectives. The perspectives of biomechanics, surgery, assessment, and communication have been examined to explore the fundamentals of the concept of subacromial impingement and to determine whether it still makes sense to use this term, in a synthesis that will be useful to clinicians in both orthopaedics and rehabilitation.

## 2. Materials and Methods

This study is a narrative review. The literature search was conducted using PubMed (https://www.nlm.nih.gov, accessed 18 July 2026) and included only English-language literature.

This work was developed as a structured narrative review rather than a formal systematic review. For this reason, no quantitative synthesis or standardised risk-of-bias assessment was conducted. The objective was to provide a clinically oriented and up-to-date synthesis of the available evidence.

The review includes systematic reviews, meta-analyses, randomised controlled trials, observational studies, narrative reviews, editorials, and clinical practice guidelines issued by recognised scientific societies.

The research was carried out using literature from the last 10 years (2016–2026), with greater emphasis placed on findings from the last 5 years and, in general, on the most recent data. In the Discussion section, sources dating from before the specified period were used when they were considered relevant and when no more recent updates on the subject were available. Four literature reviews were carried out on the four topics we decided to explore in depth:

- Pathophysiological aspects: to explore the biomechanical rationale.
- Surgical evidence: because acromioplasty is reported in some publications as a treatment option.
- Assessment tests: because clinical tests designed to provoke symptoms are still being used in studies investigating treatment options for SIS.
- Communication implications: because SIS is a term still widely used in clinical practice.

For each review, an initial selection was made based on the title, followed by a second selection based on the abstract, which determined which articles were read in full. Articles were included based on their contribution to the understanding of the anatomy, pathophysiology, diagnosis, and management of subacromial shoulder pain and rotator cuff tears in relation to the concept of subacromial impingement. Priority was given to reviews and meta-analyses. Publications containing explanations based on outdated references were excluded.

The reference lists of the selected articles were also screened to further explore topics considered relevant, which could represent a limitation. Due to the multitude of terms used in the literature to address the same topic (an issue we will address later), the ‘cited by’ and ‘similar articles’ functions of PubMed for selected articles were also utilised. This may represent a limitation and was used solely for the purpose of identifying and expanding the range of terms to be used in the research.

These topics were further explored using the following search strategies:

- Biomechanical and pathophysiological considerations: the search included the terms “rotator cuff tear”, “rotator cuff tears”, “subacromial space”, “subacromial pain”, and “subacromial impingement syndrome”, combined with keywords such as “aetiology”, “biomechanics”, “pathophysiology”, “pathomechanism”, and “kinematics” in the Title/Abstract field.
- Surgical evidence: the terms “subacromial decompression” or “acromioplasty”, with or without “rotator cuff tears”, in the Title/Abstract field.
- Clinical assessment: the search included the term “shoulder” combined with keywords such as “physical tests”, “examination tests”, or “special tests” in the Title/Abstract field.
- Communication issue: the search included the terms “rotator cuff-related shoulder pain” or “shoulder pain”, combined with keywords such as “education”, “management”, and “label” in the Title/Abstract field.

## 3. Results

### 3.1. Biomechanical and Pathophysiological Considerations

The term ‘impingement’ is commonly used in the medical literature as a causal explanation for shoulder pain and tendon injuries. The concept is based on the ‘extrinsic theory’, according to which a reduction in the subacromial space causes tendon compression, which in turn results in pain and injury. This explanation is still widely accepted as the cause of tendon injuries, but the supporting sources are often limited [12]. This reduction is thought to be due to the shape of the acromion or to a muscular imbalance in the shoulder and scapula [13,14]. However, static contact between the rotator cuff and the acromion appears to be an epiphenomenon resulting from rotator cuff insufficiency, whether due to poor muscle recruitment in an otherwise intact rotator cuff or to an existing rotator cuff tear [15,16]. Furthermore, a recent systematic review and meta-analysis showed that there is no consistent pattern regarding the correlation between acromiohumeral distance and pain or disability in participants with subacromial pain syndrome, and no consistent increase in subacromial space with improvement in pain or disability over time [17]. Regarding the acromion, it undergoes physiological age-related changes owing to calcification of the coracoacromial ligament [18], the thickness of which does not correlate with the severity of any concomitant tendon lesion [19]. There do not appear to be any clear correlations between acromial shape and the occurrence of certain conditions [20], and imaging abnormalities of the subacromial space are common in asymptomatic shoulders [21].

A further line of evidence that contradicts the extrinsic theory is that tendon lesions occur more frequently on the articular side or within the tendon substance itself, rather than on the bursal surface, where they would be expected if they resulted from mechanical abrasion against the overlying acromion [22].

An important insight into the relationship between movement, subacromial space, and potential compression of the tendon structures can be gleaned from a 2020 review [23], in which studies investigating the acromiohumeral distance during arm movement using different imaging modalities failed to support an association between pain occurring in the mid-range of motion and narrowing of the subacromial space. The rotator cuff tendon insertion is closest to the acromion between 40° and 75° of arm elevation. Beyond this range, owing to rotation of the humeral head, the tendon footprint is no longer located within the subacromial space but lies posteriorly during arm elevation and medially during abduction. In a subsequent study, it became clear that during movements beyond 90°, the supraspinatus tendon comes into contact with the posterior aspect of the glenoid cavity, away from the subacromial space, which is completely abandoned beyond 90° [24]. Consequently, according to this review and the subsequent study, there is no anatomical basis for attributing pain occurring between 70° and 120° of arm elevation—as commonly reported by patients labelled with ‘subacromial impingement’—to mechanical contact between the rotator cuff tendons and the acromion (Figure 1).

Contemporary literature consistently attributes tendon lesions to the mechanical loading experienced by the tendons, comorbidities (e.g., diabetes), lifestyle factors (e.g., cigarette smoking), genetic predisposition, friction between tendon components, and age-related degeneration [25], with particular emphasis on conditions affecting the microcirculation [26]. Associated medical conditions appear to have a significant impact on injuries in people under the age of 50 [27]. This does not mean, as we will discuss later, that subacromial pain is caused exclusively by tendon degeneration, but it suggests that the most likely mechanism underlying tissue injury (which may represent part of the painful picture) is not contact with the overlying bone.

### 3.2. Surgical Evidence

An important indication of the limited role of the acromion (i.e., acromial morphology and the height of the acromiohumeral arch) in painful shoulder conditions comes from studies investigating isolated acromioplasty, which has been shown to produce outcomes comparable to those of sham (i.e., placebo) surgery, as demonstrated by both recent high-quality randomised controlled trials [28,29] (including those with long-term follow-up [30]) and systematic reviews [31,32]. Thus, the observed clinical outcomes may be attributable to factors associated with the surgical intervention itself, rather than to acromioplasty per se, such as contextual effects, postoperative rest, and postoperative rehabilitation. Recent evidence has reinforced the concept that adding acromioplasty to rotator cuff repair results in re-tear rates and clinical outcomes comparable to those achieved with rotator cuff repair alone in studies with long-term follow-up [33,34]. Accordingly, recent reviews [35,36,37,38,39,40] have concluded that the routine addition of acromioplasty provides no benefit in preventing recurrent rotator cuff tears, which would be expected if the acromion were genuinely involved in tendon degeneration. Although limited evidence supports its use as an adjunctive procedure during rotator cuff repair [36], some authors do not intend it as a way to reduce impingement but rather to address specific intraoperative requirements in selected patients—for example, to improve exposure of the surgical field [41] or in the presence of marked calcification and thickening of the coracoacromial ligament [42]. However, the study reporting higher re-tear rates in patients with a thicker coracoacromial ligament has been criticised because of methodological bias and a low follow-up rate [43].

### 3.3. Validity of Clinical Tests

The validity of so-called “special tests” for the shoulder has been strongly called into question [44,45,46,47,48], and this further complicates the clinical assessment of shoulder pain, with validity defined as the extent to which a test actually measures what it is intended to measure. Diagnostic manoeuvres intended to identify the structure responsible for symptom generation may have the ability to reproduce symptoms, but they do not seem to have the ability to discriminate among competing diagnoses because they have low validity of the underlying pathomechanical construct [44]. For example, although Neer presented the Neer sign as a primary diagnostic tool, the subscapularis tendon actually contacts the glenoid rim before the supraspinatus tendon can contact the anteroinferior border of the acromion [45]. Consequently, the Neer sign may consistently yield a positive result in individuals with shoulder pain without identifying a particular anatomical structure. Rather than assessing impingement of the supraspinatus tendon against the coracoacromial arch, it may reproduce internal contact between the subscapularis tendon and the anterior glenoid rim. The Hawkins test provides another important example of the limitations of special tests. During this physical examination manoeuvre, the clinician theoretically reproduces impingement by passively combining shoulder internal rotation and abduction. Therefore, if tendon compression were the cause of pain, the test should reliably reproduce the symptom in patients diagnosed with subacromial impingement. Instead, it has low sensitivity and specificity [44], thereby calling into question both the theoretical basis of the test and the proposed mechanism underlying the patient’s pain, since pain typically occurs during active movement, when muscle contraction imposes tensile loading on the tendon structures.

### 3.4. Comunication Issues

When patients with a diagnosis of SIS attribute their pain to structural damage, they are also significantly more likely to choose more invasive treatments, as documented both in patient surveys [49,50,51] and in studies examining clinicians’ perceptions [52]. Patients given a predominantly biomechanical diagnosis are also significantly more likely to request further imaging [53]. However, such diagnostic investigations may themselves be harmful to patients: rotator cuff imaging is associated with a high rate of false-positive results in relation to the patient’s symptoms [54], as many rotator cuff lesions represent natural, asymptomatic degenerative changes. Patients who have not been adequately educated may nevertheless associate these abnormalities with their symptoms, undergo unnecessary treatment for clinically irrelevant lesions, and experience greater concern about their condition. Patients frequently have a strong preference for imaging [55] because they commonly regard their problem as biomechanical and wish to identify its cause [56], despite guidelines advising against imaging during the first few weeks for the reasons outlined above [57].

## 4. Discussion

### 4.1. Surgical Implications

With regard to surgical options, current clinical practice guidelines [40], including those issued by the Italian Society of Shoulder and Elbow Surgery (SICSeG) in 2021 [58] and the American Academy of Orthopaedic Surgeons (AAOS) in 2025 [59], do not recommend isolated acromioplasty as a surgical procedure aimed at decompressing the subacromial space. The routine addition of acromioplasty seems to provide no benefit in preventing recurrent rotator cuff tears. Beyond these technical considerations, which should remain individualised according to the surgical context, it is evident from the literature on surgery that the classical tendon–acromion compression model is not adequately supported as a sufficient or predominant causal explanation for the clinical presentation historically labelled as subacromial impingement syndrome.

### 4.2. Clinical Implications

Regarding assessment tests, and, in particular, the difference in the way symptoms are triggered during active and passive movement, it is active tension rather than compression mechanisms that is thought to activate mechanosensitive nociceptors—sensitised by overload, degenerative lesions, or inflammatory processes—generating a neural signal that may give rise to pain when the imposed load is interpreted as exceeding the tissue’s capacity to tolerate it [47].

More fundamentally, the special tests are inherently aimed at measuring the construct of pain through mechanical manoeuvres, although pain is, by definition, an emergent property. Indeed, pain arises from the non-linear interaction of structural, biological, psychological, and social variables. The structure responsible for symptom generation therefore cannot always be identified accurately using resisted tests alone, which should represent only one component of the overall clinical assessment [60,61]. These tests therefore have a weak anatomical rationale and are characterised by substantial inter-examiner variability in their administration, resulting in marked heterogeneity and precluding aggregation of the findings. Shoulder disorders may also involve a bursal inflammatory response that alters the mechanical assumptions underlying the tests, thereby confounding the interpretation of their results and potentially leading to numerous false-positive results. Hence, such tests cannot reliably identify the target structure, because each movement activates and deforms multiple structures within the shoulder complex [45], making it impossible to isolate a single structure during either passive or active movement [46,62,63]. According to some authors, the special tests commonly used to establish the presence of subacromial impingement cannot reliably confirm the condition, as they do not provide sufficient diagnostic information to precisely identify the structure responsible for symptom generation, making any positive test result inherently non-specific [45,64], except when used to rule out a massive rotator cuff tear [48,64]. When appropriate, clinicians may use these tests as pain-provocation tests to guide the direction of therapeutic exercise. They may also remain useful as a means of communication among clinicians.

### 4.3. Communicative and Management Implications

Numerous studies have highlighted the adverse consequences of an inaccurate biomechanical explanation. Such an explanation may lead patients to believe that elevating the arm causes harmful friction between the tendon and the overlying bone. This belief may lead them to avoid arm elevation, thereby contributing to kinesiophobia, relative deconditioning, and even increased pain. This does not mean that the use of the term ‘subacromial impingement’ automatically leads to a worsening of the patient’s symptoms; however, in light of the above considerations, and given the real possibility that impingement may not contribute to subacromial pain, it is potentially harmful to the patient to attribute their pain solely to a biomechanical explanation—one which, moreover, is highly questionable.

If imaging is performed, the physician and/or therapist are responsible for educating the patient prior to the examination about the possibility that any findings may not correlate with the clinical presentation and should then help the patient interpret the results [65]. For these reasons, the healthcare team should consider diagnostic labels, special tests, and imaging findings as potential sources of nocebo effects in the absence of effective clinician–patient communication [66]. As stated by Reeve et al. in their 2026 systematic review of patient experiences of diagnostic uncertainty in musculoskeletal care: “Successful communication and a strong clinical relationship can facilitate shared decision-making throughout the diagnostic process, and help mitigate the potentially disempowering effects of uncertainty” [67]. Without such prior education, patients may be less likely to consider conservative management, even though 2026 guidelines recommend it as the first-line approach for subacromial shoulder pain [68]. In addition to being effective in a substantial proportion of patients, conservative management is associated with lower costs and risks and less time away from work, without precluding surgery at a later stage.

### 4.4. Terminology Proposals

The need for a conceptual revolution, before a terminological one, arises from recognition of the importance of the diagnoses used by healthcare professionals to categorise patients, both for the advancement of knowledge and for clinical practice. Multiple names for a single clinical entity hinder data aggregation, impair communication among different professional groups, and undermine secondary research. Although a body of research on shoulder pain challenges the existence of subacromial impingement, dozens of articles continue to be published each year on the treatment of patients enrolled in studies on the basis of the special tests discussed above.

Several terms have been proposed to describe this clinical presentation and replace the term ‘subacromial impingement’ (Table 1). In 2014, Diercks et al. [69] proposed the diagnostic label ‘subacromial pain syndrome’ (SAPS). They defined it as an umbrella term encompassing non-traumatic, usually unilateral shoulder disorders characterised by pain localised around the acromion and aggravated during or after arm elevation. The label encompasses several clinical and radiological entities, such as bursitis, calcific tendinopathy, supraspinatus tendinopathy, partial-thickness rotator cuff tear, long-head biceps tendinopathy, and rotator cuff degeneration. Although it encompasses heterogeneous clinical presentations, these conditions share anterior shoulder pain as a common feature. In 2025, Zadro et al. [70] suggested that terms such as ‘shoulder sprain’, ‘bursitis’, or ‘an episode of shoulder pain’ may sound simpler and less concerning to patients. However, in light of the issues associated with imaging and nocebo effects, these terms may respectively evoke the idea of traumatic tissue damage, imply that inflammation is solely responsible for the symptoms, or leave patients uncertain and encourage them to seek imaging. Chris Littlewood has proposed the use of the term ‘rotator cuff tendinopathy’ in clinical practice [71]. This label may be acceptable because patients generally recognise that tendons can undergo age-related degeneration in adulthood. It may also be less alarming than the idea that the tendon is repeatedly abraded against the acromion during every episode of arm elevation, and it is credible because the disorder predominantly affects middle-aged individuals, who may understand tendon ageing in the same way as skin wrinkling or greying hair. Patients are also likely to have direct or indirect experience of tendinopathy in other body regions, often with a favourable outcome. Nevertheless, the term that appears most appropriate is ‘rotator cuff-related shoulder pain’, proposed by the British physiotherapist Jeremy Lewis [7], as it identifies the structures associated with the disorder without necessarily attributing the condition to a single causal mechanism. This label also encompasses shared management principles—education, desensitisation, and therapeutic exercise—and a shared prognosis, with satisfactory symptom reduction within 12 weeks. In any case, as suggested by Zadro et al. [70], clinicians should consider not only the terminology itself but also their patients’ cultural and linguistic backgrounds: choosing terminology appropriate to the patient’s social and cultural context can strengthen the therapeutic alliance. Adapting diagnostic language so that it is culturally sensitive and less pathologising may help mitigate potentially harmful effects and ensure that all patients receive appropriate, non-threatening explanations consistent with their cultural context.

## 5. Conclusions

Although the term subacromial impingement remains widely used in both clinical practice and research, the international specialist literature, drawing on surgical, diagnostic, and prognostic evidence, is increasingly moving away from the concept of tendon–acromion impingement as the main cause of pain in the subacromial region. Although a clear consensus about all the causal mechanisms of shoulder pain and the terminology to be used has not yet been reached, the term ‘subacromial impingement’ appears to be a flawed biomechanical explanation and may be misleading for the patient; for these reasons, it should be discontinued. Physicians and physiotherapists should provide patients with prior education regarding the factors that contribute to shoulder pain and guide them in decisions concerning imaging and in the interpretation of imaging findings, using the least pathologising and least causally presumptive label that remains clinically informative. It is hoped that an increasing number of publications and scientific societies will further investigate the nature of subacromial pain and work towards selecting and disseminating appropriate medical terminology in order to promote change in current clinical practice.

## 6. Limitations

Due to the great heterogeneity of terms used in the literature to define the same problem, we searched the reference lists of the most relevant articles identified in our research. We acknowledge that no formal systematic review protocol, PRISMA flow, or risk-of-bias tool was applied. Only articles in English were included.

## Figures and Tables

**Figure 1:**
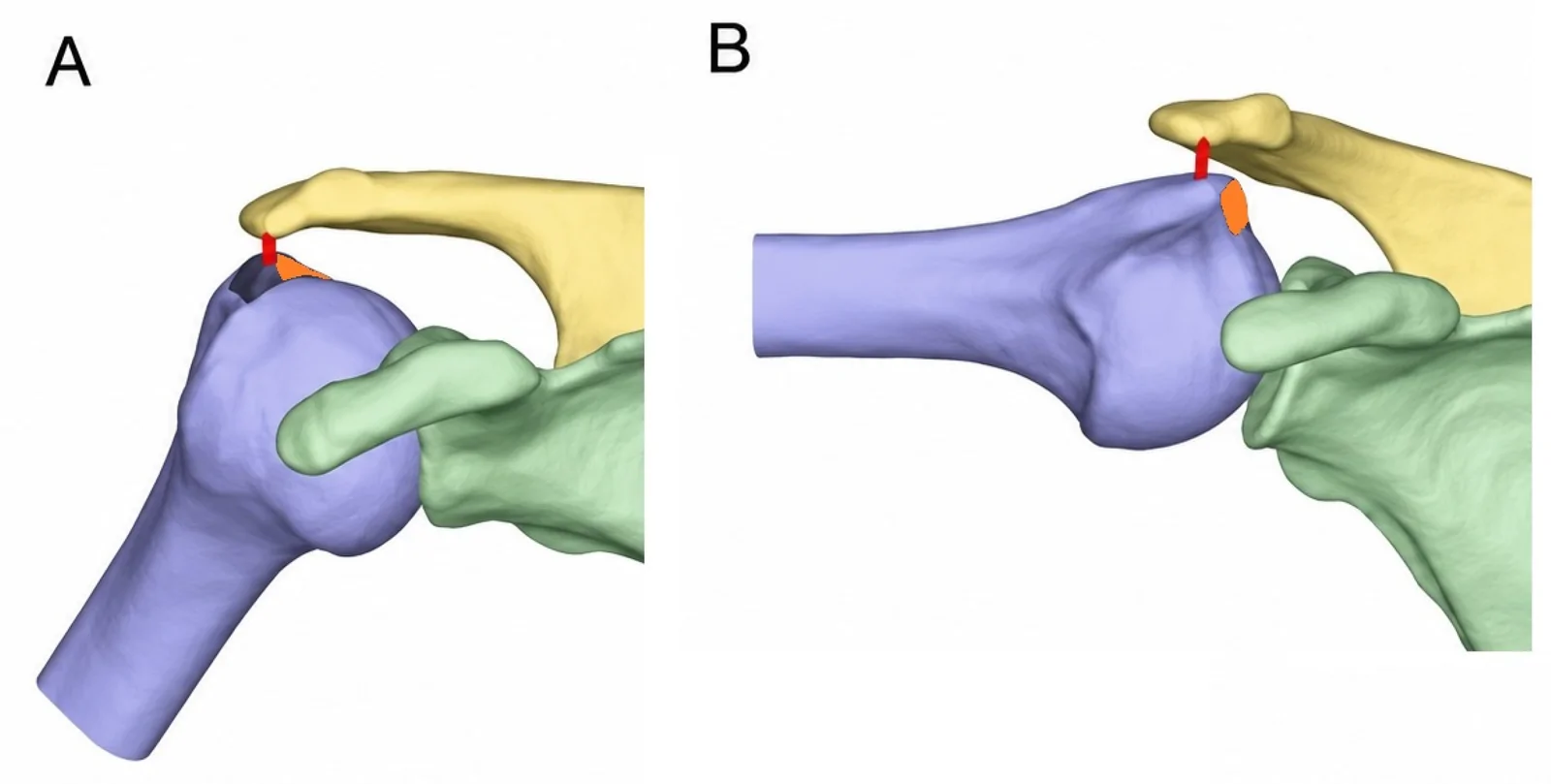
During abduction, the subacromial space decreases, but the tendon insertion clears the space. (**A**) the insertion of the rotator cuff tendons lies beneath the acromion when the humerus is adducted. (**B**) the insertion of the tendons no longer lies beneath the acromion during abduction.

**Table 1:** Alternative Terminology for “Subacromial Impingement Syndrome”.

Proposed Terminology	Subacromial Pain Syndrome	Rotator Cuff Tendinopathy	Rotator Cuff-Related Shoulder Pain
Pathoanatomical specificity	No specificity	Localised, but confined solely to the tendon	Comprehensive and accurate: it identifies the rotator cuff as the main cause of the condition, but not as the sole factor
Clinical utility	Useful for communication between professionals in cases of clinical uncertainty	Useful in clinical practice	Useful for literature and communication among healthcare professionals
Patient acceptability	The definition is too vague for the patient	Understandable and acceptable to the patient	Difficult for the patient to understand
Prognostic value	Low predictive value	It helps to determine the treatment strategy: initial conservative management, focusing on pain desensitisation and therapeutic exercise	This label encompasses shared management principles—education, desensitisation, and therapeutic exercise—and a shared prognosis, with satisfactory symptom reduction within 12 weeks
Nocebo potential	Terminology that is too vague can have nocebo effects on patients because of the uncertainty it leaves them with	Identifying the tendon alone as the cause of pain could lead the patient to focus solely on the structural issue	It may be difficult for the patient to understand
Advantages	An umbrella term that promotes consistency among healthcare professionals	A useful label in clinical practice because it is credible, acceptable, and unlikely to cause undue concern	A technically more accurate term because it identifies the structures associated with the disorder without necessarily attributing it to a single causal mechanism

## Data Availability

The original contributions presented in this study are included in the article. Further inquiries can be directed to the corresponding author.
